# A New Interesting Moth Lacewing (Neuroptera: Ithonidae) from the Mid-Cretaceous Kachin Amber †

**DOI:** 10.3390/insects17010014

**Published:** 2025-12-22

**Authors:** Yuying Li, Siting Liu, Shumin Li, Jie Yang, Chaofan Shi, Dong Ren, Qiang Yang

**Affiliations:** 1 South China Biodiversity Research Center, School of Life Sciences, Guangzhou University, #230 Waihuanxi Road, Guangzhou Higher Education Mega Center, Guangzhou 510006, China; 2112414036@e.gzhu.edu.cn (Y.L.); lsting@e.gzhu.edu.cn (S.L.); 13724413430@163.com (J.Y.); 2 School of Earth Sciences and Engineering, Sun Yat-sen University, Guangzhou 510275, China; lishm36@mail2.sysu.edu.cn; 3 College of Life Sciences, Capital Normal University, Xisanhuanbeilu 105, Haidian District, Beijing 100048, China; rendong@mail.cnu.edu.cn

**Keywords:** Ithonidae, Neuroptera, Kachin amber, Cretaceous, fossil

## Abstract

One new genus and species of Ithonidae, *Cretithone zhangi* gen. et sp. nov., is described from the mid-Cretaceous Kachin amber. *Cretithone* gen. nov. is distinguished from other fossil ithonids by the combination of the following characteristics: exceptionally broad costal space; proximal branches of RP fused into a single vein and then fused with M; medial branches of RP fused with each other; simple basal M, with marginal forks; CuA divided into two main branches, the anterior branch with posteriorly directed subbranches and the posterior branch with anteriorly directed subbranches; and most proximal branches of CuP fused with adjacent branches. The new genus and species has been assigned to the *Principiala* genus-group by the fusion of the proximal branches of RP and the peculiar configuration of M and CuA. Variations in the forewing characteristics among the fossil ithonids are briefly discussed.

## Simple Summary

As a relict family within Neuroptera, Ithonidae sensu lato exhibit a relatively rich fossil record. However, severely underrepresented Cretaceous diversity (especially during the Late Cretaceous) and the lack of fossil calibration for key traits impede the comprehensive understanding of Ithonidae’s evolutionary history. This study describes one new genus and species from the mid-Cretaceous Kachin amber, *Cretithone zhangi* gen. et sp. nov., and assigns it to the *Principiala* genus-group. *Cretithone* gen. nov. shows a closer systematic position to *Phyllithone* Liu, Lu, Wang, Xu and Zhuo, 2025, as evidenced by shared forewing characteristics.

## 1. Introduction

Ithonidae sensu lato is a relict family of Neuroptera, comprising 10 genera with 45 extant species and 28 genera with 46 extinct species [1,2,3]. Adults of Ithonidae s.l. are characterised by a robust body, head more or less retracted under the pronotum, long and broad antehumeral space in both wings, recurrent humeral veinlet, and the presence of distal nygmata [4]. Within Neuroptera, it’s widely accepted as a sister to Myrmeleontiformia and as having a close relationship with Psychopsoidea [5,6,7].

Intrafamilial relationships of Ithonidae s.l. remain inadequately resolved, particularly concerning fossil taxa. Based on total-evidence phylogenetic analysis, Ithonidae s.l. was previously recovered as a monophyletic clade with three lineages: Ithonidae sensu stricto (moth lacewings), Polystoechotidae (giant lacewings), and Rapismatidae (montane lacewings) [4]. Subsequently, Zheng et al. further refined Ithonidae’s morphological classification and divided the family into three genus-groups—the *Ithone* genus-group, the *Polystoechotes* genus-group, and the *Rapisma* genus-group [8,9]. Further taxonomic revision occurred when Lu et al. formally established the *Principiala* genus-group as an independent unit, which was originally suggested by Makarkin and Archibald [10,11]. Additionally, Makarkin et al. proposed the provisional establishment of the subfamily Epigambriinae under Ithonidae s.l., motivated by the unique morphological traits of its constituent genera, while deliberately segregating these taxa from undetermined nominal lineages [12]. However, the severely underrepresented Cretaceous diversity (especially during the Late Cretaceous) and the lack of fossil calibration evolution for key traits impede the comprehensive phylogenetic reconstruction of Ithonidae s.l. [2]. Thus, newly discovered ithonid fossils from the Cretaceous are crucial for addressing these gaps.

Currently, only nine ithonid species have been documented from the Cretaceous; two were reported from the Kachin amber, *Burmithone pennyi* Lu, Zhang, Ohl and Liu, 2017 and *Phyllithone dongshengi* Liu, Lu, Wang, Xu and Zhuo, 2025 [1,10]. Herein, we describe one new genus and species of Ithonidae from the mid-Cretaceous Kachin amber, expanding both the species richness and morphological diversity of Ithonidae s.l. preserved in amber.

## 2. Materials and Methods

This study is based on two specimens from Myanmar amber. The amber pieces were collected from the Hukawng Valley (the state of Kachin in northern Myanmar). The map of the Hukawng Valley was provided by Grimaldi et al. [13]. The volcaniclastic matrix of the amber is estimated to be approximately 98.79 ± 0.62 Ma, i.e., earliest Cenomanian, near the Albian/Cenomanian (Early/Late Cretaceous) boundary [14]. The biological inclusions of Kachin amber represent a sample of a tropical forest community in equatorial southeastern Asia at approximately 12.4 °N palaeolatitude [13,15,16,17,18]. The specimens were deposited in the collections of the Key Laboratory of Insect Evolution & Environmental Changes, College of Life Sciences, Capital Normal University, Beijing, China (CNUB; Dong Ren, Curator). The specimens were examined using a Zeiss Discovery V20 (Carl Zeiss, Oberkohen, Germany) and a Nikon SMZ1270 stereomicroscope (Nikon corporation, Tokyo, Japan). Photographs were taken using an AxioCam HRc and an iMG SC600C digital camera (Carl Zeiss, Oberkohen, Germany) attached to the Zeiss Discovery V20 and Nikon SMZ1270 stereomicroscope. Line drawings were prepared with Adobe Illustrator CC 2020.

Venational terminology, in general, follows Kukalová-Peck and Lawrence [19] as interpreted by Yang et al. [20,21] and Oswald [22].

## 3. Results

*Systematic palaeontology*

Class Insecta Linnaeus, 1758

Order Neuroptera Linnaeus, 1758

Family Ithonidae Newman, 1853 sensu Winterton and Makarkin, 2010

Genus *Cretithone* Li, Shi, Ren and Yang gen. nov.

Type species: *Cretithone zhangi* Li, Shi, Ren and Yang gen. et sp. nov.

Etymology. The generic name is a combination of the Latin *Cret-* (meaning Cretaceous), and *ithone* (a genus-group name of Ithonidae), in reference to the age of the holotype’s deposit. Gender feminine.

Diagnosis. Humeral veinlet recurrent with multiple branches; trichosors present along the entire wing margin; costal space exceptionally broad; costal gradate series of crossveins incomplete, regularly arranged; proximal branches of RP fused into a single vein and then fused with M; medial branches of RP fused with each other; basal M simple, with marginal forks; CuA divided into two main branches, the anterior branch with posteriorly directed subbranches and the posterior branch with anteriorly directed subbranches, forming a subtriangular area; and most proximal branches of CuP fused in forewing.

Remarks. *Cretithone* gen. nov. is assigned to Ithonidae based on the head being completely retracted under pronotum, humeral veinlet strongly recurrent with multiple branches, exceptionally broad costal space at proximate portion, and numerous, irregular crossveins.

This new genus can be distinguished from the other fossil genera of Ithonidae by the combination of the following characteristics: (1) costal gradate series of crossveins incomplete, regularly arranged (absented in the majority of Ithonidae genera, except *Allorapisma* Makarkin and Archibalda, 2009, *Frustumopsychops* Khramov in Ponomarenko et al., 2014, *Palaeopsychops* Andersen, 2001, and *Principiala* Makarkin and Menon, 2007); (2) distal parts of ScP fused with RA, ScP+RA reaching anterior margin before wing apex (distal parts of ScP and RA separated in most genera of the *Ithone* genus-group and the *Rapisma* genus-group; termination position of ScP+RA bent posteriad in most genera of the *Polystoechotes* genus-group and *Rapisma* genus-group); (3) proximal branches of RP fused into a single vein and then fused with M (the fusion absent among most Ithonidae genera; proximal branches of RP fused with adjacent branch but not fused with M in most genera of the *Principiala* genus-group; at least six proximal branches of RA, respectively, fused with M in *Phyllithone* Liu, Lu, Wang, Xu and Zhuo, 2025); (4) medial branches of RP fused (the fusion absent in most genera of Ithonidae except *Phyllithone*); (5) M only branched distally (in most Ithonidae genera, M separated into MA and MP, with the exception of the *Principiala* genus-group); (6) CuA divided into two main branches, the anterior branch with posteriorly directed subbranches and the posterior branch with anteriorly directed subbranches (CuA branched posteriad in most genera of Ithonidae except the *Principiala* genus-group); and (7) most proximal branches of CuP fused (such fusion absent in the other genera of Ithonidae) [1,8,11,23,24,25].

*Cretithone zhangi* Li, Shi, Ren and Yang gen. et sp. nov.

**Figure 1:**
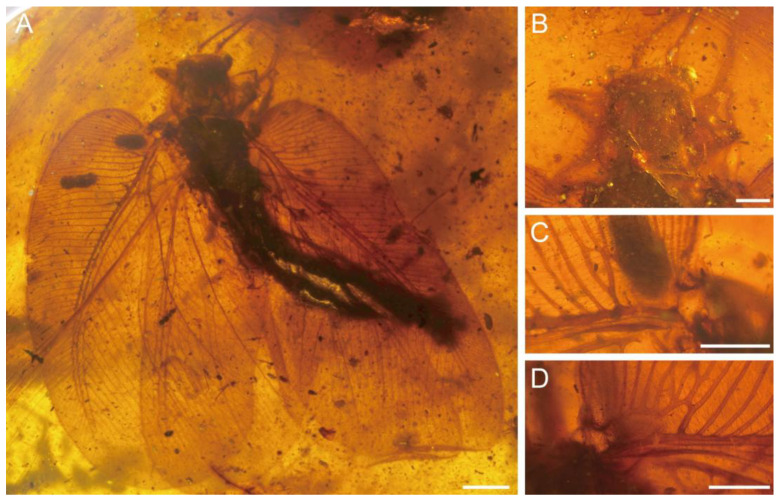
Photographs of *Cretithone zhangi* gen. et sp. nov., holotype CNU-NEU-MA2018145. (**A**) Holotype in dorsal view; (**B**) head in dorsal view; (**C**) humeral veinlet of left forewing; and (**D**) humeral veinlet of right forewing. Scale bars: (**A**) 2 mm and (**B**–**D**) 1 mm.

**Figure 2:**
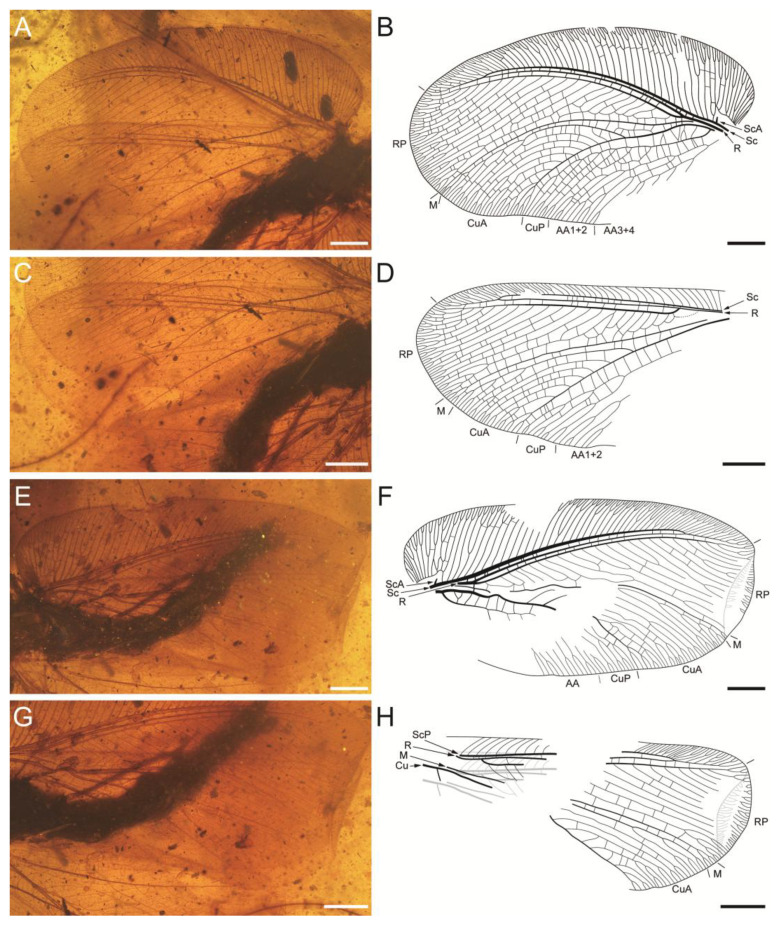
Photographs and line drawings of *Cretithone zhangi* gen. et sp. nov., holotype CNU-NEU-MA2018145. (**A**,**B**) Left forewing; (**C**,**D**) left hind wing; (**E**,**F**) right forewing; and (**G**,**H**) right hind wing. Scale bars: 2 mm.

**Figure 3:**
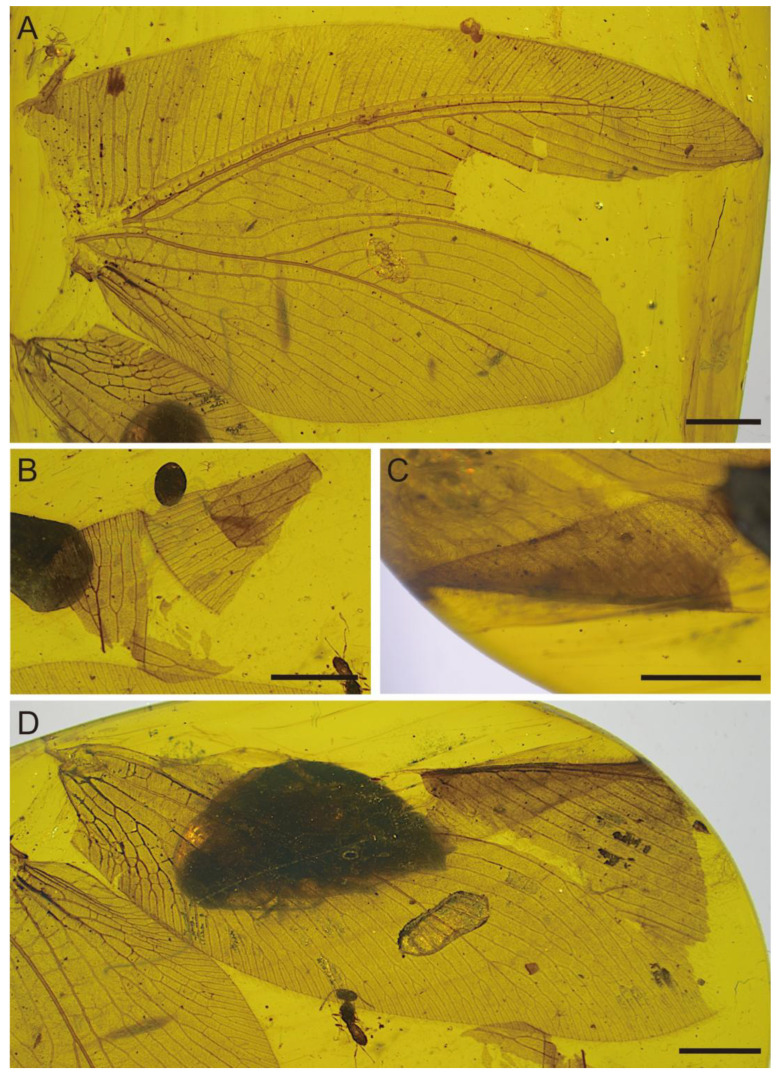
Photographs of *Cretithone zhangi* gen. et sp. nov., paratype CNU-NEU-MA2018101. (**A**) Left forewing; (**B**) left forewing fragment; (**C**) the folded region of the right forewing; and (**D**) right forewing. Scale bars: 2 mm.

**Figure 4:**
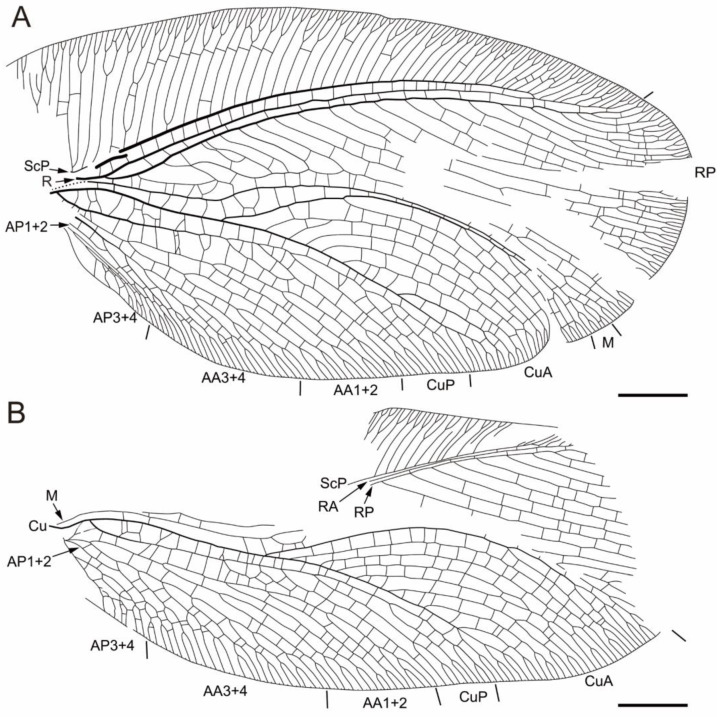
Line drawings of *Cretithone zhangi* gen. et sp. nov., paratype CNU-NEU-MA2018101. (**A**) Left forewing; (**B**) right forewing. Scale bars: 2 mm.

Figure 1, Figure 2, Figure 3 and Figure 4

Material. Holotype: CNU-NEU-MA2018145. Paratype: CNU-NEU-MA2018101.

Etymology. The specific epithet honour of Mr. Xinfang Zhang (curator of the Ningshiguang Amber Museum) for donating the type specimen in this study.

Locality and horizon. Hukawng Valley, Kachin State, northern Myanmar; lowermost Cenomanian, Upper Cretaceous.

Diagnosis. As for the genus.

Description. Holotype (Figure 1 and Figure 2). Gender unknown. Body robust; head (Figure 1B) completely retracted under pronotum; compound eyes globular; antenna filiform.

Left forewing oval (Figure 2A,B), ca. 18.72 mm long and 10.44 mm wide as preserved (measured at widest part); right forewing (Figure 2E,F) ca. 18.90 mm long and ca. 9.72 mm wide as preserved. Forewing covered with dense short hairs. Trichosors prominent along wing apex, weakly along posterior margins; nygmata not detected. Humeral veinlet (Figure 1C,D) strongly recurrent and branched, with a protruding projection. Costal space exceptionally broad near the wing base, narrowed apically; ca. 51 (left forewing/LFW) or 50 (right forewing/RFW) subcostal veinlets (including branches of humeral veinlet) preserved, most subcostal veinlets forked near costal margin; costal gradate series of crossveins incomplete but regularly arranged. Sc divided into ScA and ScP in humeral area; ScA short; ScP stout, ending curved and fused with RA; termination position of ScP+RA bent anteriad, reaching anterior margin before wing apex; ScP+RA pectinately forked anteriad, with 6 (LFW) or 7 (RFW) branches dichotomously forked near wing margin. R stout, divided into RA and RP near wing base; the width of RA space narrow, approximately equal to that of subcostal space; RP pectinately forked, with 20 (LFW) or 23 (RFW) branches; 6 most proximal branches of RP fused into a single vein, which then fused with M; medial 3 branches of RP fused into one, distally branched; RP11 and RP12 fuse into a single vein (RFW); similarly, RP14 and RP15 form another single vein (RFW); other RP branches forked near wing margin except RP10 (LFW). M simple, without primary fork, dichotomously branched near wing margin. Cu stout, divided into CuA and CuP near wing base; CuA stout, divided into two main branches at approximately one-fifth (LFW) or one-third (RFW) of its length from base; CuA1 with many posteriorly directed subbranches, CuA2 with many anteriorly directed subbranches, forming a subtriangular area; an intercalary vein present between M and CuA, originating from CuA stem and distally fused with CuA1; proximal 2 subbranches of CuA2 fused with CuA1 (LFW); most subbranches of CuA1 and CuA2 forked near wing margin; numerous cua-cup crossveins preserved, 2cua-cup, 3cua-cup, and 4cua-cup (LFW) or only 1cua-cup (RFW) branched. CuP pectinately forked at approximately one-fourth of its length from base, with 6 branches; 3 most proximal branches of CuP fused together, forming 2 long cells; several branches of CuP forked near hind margin. Anal vein incompletely preserved. AA divided into AA1+2 and AA3+4 near wing base; AA1+2 dichotomously forked distally, with additional forks; AA3+4 pectinately forked posteriad. Forewing with numerous irregularly placed crossveins.

Left hind wing (Figure 2C,D) ca. 14.61 mm long as preserved, ca. 7.81 mm wide as preserved (measured at widest part); right hind wing (Figure 2G,H) ca. 14.22 mm long and ca. 6.67 mm wide as preserved. Trichosors prominent along the wing apex and posterior margin, but not detected along the anterior margin. Humeral and nygmata not preserved. Costal space near base considerably narrower than the corresponding region in forewing; ca. 38 (left hind wing/LHW) or 22 (right hind wing/RHW) subcostal veinlets (including branches of humeral veinlet) preserved, with a minority exhibiting branching. ScA not preserved; ScP ending curved and fused with RA; ScP+RA pectinately forked anteriad, with 7 (LHW) or 6 (RHW) branches, most of which bifurcate further. R divided into RA and RP near wing base; RP pectinately forked, ca. 21 (LHW) or 16 (RHW) branches preserved; 5 most proximal branches of RP fused into a single vein, which then fused with M; RP6 fused with M; medial 3 branches of RP fused (LHW); other branches of RP forked near wing margin. M without primary fork, branched near wing margin. CuA divided into two main branches; CuA1 with many posteriorly directed subbranches, CuA2 with many anteriorly directed subbranches; medial 2 subbranches of CuA2 fused (RHW); most subbranches of CuA1 and CuA2 forked near wing margin. CuP pectinately forked, with 4 branches; few CuP branches forked near hind margin. Anal veins incompletely preserved. Hind wing with numerous irregularly placed crossveins.

Paratype (Figure 3 and Figure 4). Gender unknown. Left forewing oval (Figure 3A,B and Figure 4A), ca. 18.61 mm long as preserved, ca. 10.58 mm wide as preserved (measured at widest part); right forewing (Figure 3C,D and Figure 4B) ca. 16.78 mm long and ca. 8.05 mm wide as preserved. Forewing covered with dense short hairs. Trichosors prominent along wing apex, weakly present along costal and posterior margins. Humeral veinlet and ScA not preserved. Nygmata not detected. Proximal portion of costal space very broad, narrowed apically; at least 40 subcostal veinlets preserved, most of them inclined toward wing apex, more strongly oblique in its apical portion, most subcostal veinlets forked near costal margin; costal gradate series of crossveins incomplete but regularly arranged. ScP stout, ending curved and fused with RA; termination position of ScP+RA bent anteriad, reaching anterior margin before wing apex; ScP+RA pectinately forked, with eight branches, several of which have additional forks; subcostal space very narrow, with irregular crossveins. R stout, divided into RA and RP near wing base; the width of RA space is approximately equal to that of subcostal space; RP pectinately forked, with 21 branches; RP2 branched near RP stem; RP3 and RP4 closely spaced, seemingly fused, connected by a crossvein; RP1 and the posterior branch of RP2 distally fused and then fused with M, the anterior branch of RP2 fused with 4 medial branches of RP, formed 6 long cells; other branches of RP forked near wing margin. M weak, without primary fork, sharply curved anteriad at approximately one-fourth of its length from the basal, branched near wing margin; numerous rp-m crossveins preserved, 5rp-m branched. Cu stout, divided into CuA and CuP near wing base; numerous m-cua crossveins preserved, 8m-cua branched; CuA stout, divided into two main branches at approximately one-third of its length from base; CuA1 with many posteriorly directed subbranches, CuA2 with many anteriorly directed subbranches and several posteriorly directed subbranches near wing margin, forming a subtriangular area; proximal 2 subbranches of CuA2 (LFW) or medial 2 subbranches (RFW) of CuA2 fused; most subbranches of CuA1 and CuA2 forked near wing margin. CuP pectinately forked at approximately one-third of its length from the basal, with 7 branches; CuP1 fused with CuP2 (LFW), or 3 most proximal branches of CuP fused together (RFW); several branches of CuP forked near hind margin; numerous crossveins in cubital space preserved, several of which forked.

Anal vein incompletely preserved. AA divided into AA1+2 and AA3+4 near wing base; AA1 and AA2 dichotomously forked into three branches, respectively; each branch ends in a marginal fork. AA3+4 pectinately forked at approximately one-fifth of its length from base, with 5 (LFW) or 7 (RFW) branches, several with additional dichotomous forks; AP1+2 short, fused with AA3+4. AP3+4 dichotomously forked; several forks of AP3+4 with additional dichotomous forks. Forewing with numerous irregularly placed crossveins.

Remarks. The primary distinctions between the holotype and paratype are as follows: (1) the number of fused proximal branches in RP (proximal 5 or 6 branches of RP fused into a single vein in holotype; RP1 and the posterior branch of RP2 distally fused in paratype); (2) the number of fused medial branches in RP (medial 3 branches of RP fused into a single vein in holotype; the anterior branch of RP2 fused with 4 medial branches of RP in paratype); (3) the presence of an intercalary vein (an intercalary vein present between M and CuA, originating from CuA stem and distally fused with CuA1 in holotype; absent in paratype); and (4) the fusion patterns of CuA (proximal 2 subbranches of CuA2 fused with CuA1 in forewing in holotype; such fusion absent in paratype).

Although there are some morphological differences in the wing characteristics between the two specimens, their similar wing shape and the fundamental configuration of the wing venation support the current classification of the two specimens as the same species.

## 4. Discussion

The systematic placement of *Cretithone* gen. nov. within the *Principiala* genus-group is established through a combination of diagnostic exclusions and positive synapomorphies. This new genus is excluded from *Ithone*, *Polystoechotes*, and *Rapisma* genus-groups by the distal fusion of ScP and RA, anteriorly bent termination position of ScP+RA, and very shallowly forked M [8]. Conversely, the assignment to the *Principiala* genus-group is supported by shared derived characteristics: the fusion of several RP branches and peculiar configuration of M and CuA [10,11]. Given current limitations in resolving deep-node relationships within Ithonidae, this morphological incompatibility with existing genus-group frameworks underscores the necessity of future phylogenetic revisions.

The discovery of the new species augments the deep-time diversity of the *Principiala* genus-group. This genus-group is exclusively restricted to fossil species from the Cretaceous and Paleogene, with the oldest record being the Early Cretaceous *Principiala rudgwickensis* Jepson, Makarkin and Jarzembowski, 2009, forming *Principiala* genus-group’s Early Cretaceous representatives together with *Principiala incerta* Makarkin and Menon, 2007 and *Principiala sinuijuensis* Won, Ri, Ri and So, 2023 [25,26,27]. Previously, only two Late Cretaceous species (*Burmithone pennyi* and *Phyllithone dongshengi*) were known; this study elevates the Late Cretaceous record to three genera and three species. All currently known Late Cretaceous records of this genus-group derive from Kachin amber [1,10]. For the Cenozoic period, the *Principiala* genus-group is represented solely by *Allorapisma chuorum* Makarkin and Archibald 2009 [11].

Wing morphological comparison across these taxa reveals that the proximal branches of RP are fused, M is simple, and the peculiar configuration of CuA (both primary branches of CuA form an equal angle with the common stem of CuA, the anterior branch has posteriorly directed subbranches and the posterior branch has anteriorly directed subbranches) constitute key diagnostic characteristics within the *Principiala* genus-group [10,11]. *Cretithone* gen. nov. can easily be distinguished from the other genera of the *Principiala* genus-group by the following characteristics: (1) costal gradate series of crossveins incomplete but regularly arranged (absented in *Burmithone* Lu, Zhang, Ohl and Liu, 2017, *Phyllithone* and *Sinuijuala* So and Won, 2022); (2) distal parts of ScP fused with RA (distal parts of ScP and RA separated in *Allorapisma*); (3) proximal branches of RP fused into a single vein, which then fused with M (at least six proximal branches of RA, respectively, fused with M in *Phyllithone*); (4) fusion of the medial branches of RP (the fusion absent in most genera of the *Principiala* genus-group except *Phyllithone*); and (5) most proximal branches of CuP fused with adjacent branches (such fusion absent in the other genera of the *Principiala* genus-group) [1,10,11,25,26,27,28]. These diagnostic characteristics further corroborate its status as a distinct genus within the genus-group. Based on shared characteristics of the wing shape, exceptionally broad costal space, RP basal branch fusion with M, and dense crossveins, *Cretithone* and *Phyllithone* are inferred to have a closer systematic relationship among *Principiala* genus-group, while these two genera are distinguished by a regular costal gradate series of crossveins, the presence of trichosors, and CuP basal branch fusion [1].

Divergent from other fossil ithonids throughout geological history, the shared wing morphology of *Cretithone* and *Phyllithone* likely suggests a unique morphotype from the Cretaceous Burmese amber Ithonidae. Their broad costal space reaches up to 4.5 times the combined width of the subcostal space and RA space, resulting in a large, approximating oval, wing profile with a length-to-width ratio (length/width) < 2.0 [1]. This also contrasts sharply with the sympatric, small-bodied fossil ithonid, *Burmithone* (length-to-width ratio > 2.12) [10], reflecting pronounced morphological disparity that may imply distinct functional adaptations and potential ecological niche differentiation in the Late Cretaceous ecosystems. These hypotheses require further validation.

## 5. Conclusions

One new genus and species, *Cretithone zhangi* gen. et sp. nov. is described from mid-Cretaceous Kachin ambers. This new species enriches the diversity of amber members reported in this area and provides a new understanding of the morphology and characteristics of Ithonidae.

## Data Availability

The original contributions presented in this study are included in the article. Further inquiries can be directed to the corresponding authors.

## Abbreviations

AA, anterior analis; AP, posterior analis; CuA, anterior cubitus; CuP, posterior cubitus; M, media; MA, anterior media; MP, posterior media; RA, anterior radius; RP, posterior radius; RP1, proximal-most branch of RP; RP2, branch of RP distad RP1; Sc, subcostal; ScA, subcosta anterior; and ScP, posterior subcosta.
